# Psychological assessment and the role of the psychologist in early palliative care

**DOI:** 10.3389/fpsyg.2024.1437191

**Published:** 2024-11-13

**Authors:** Pamela Iannizzi, Alessandra Feltrin, Rosalba Martino, Chiara De Toni, Antonella Galiano, Ardi Pambuku, Mariateresa Nardi, Nicla Meraviglia, Antonella Brunello, Vittorina Zagonel

**Affiliations:** ^1^Hospital Psychology, Veneto Institute of Oncology IOV – IRCCS, Padua, Italy; ^2^Department of Oncology, Medical Oncology 1, Veneto Institute of Oncology IOV – IRCCS, Padua, Italy; ^3^Pain Therapy and Palliative Care Unit, Veneto Institute of Oncology IOV – IRCCS, Padua, Italy; ^4^Clinical Nutrition Unit, Veneto Institute of Oncology IOV – IRCCS, Padua, Italy

**Keywords:** advanced cancer patients, early palliative care, psycho-oncology, psychological assessment, share care planning

## Abstract

**Background:**

Early palliative care (EPC) is a recommended model for improving the quality of life for patients with advanced cancer and their caregivers. However, limited research has focused on the role of psychological evaluation within EPC. The Veneto Institute of Oncology (IOV), a Comprehensive Cancer Centre, employs an interdisciplinary team to assess patients with advanced-stage disease. This study aims to assess the psychological needs of these patients, investigate any correlations between psychological symptoms (PSs) and factors such as awareness of diagnosis and prognosis, symptoms detected using the Edmonton Symptom Assessment System (ESAS), as well as the patient’s gender, age, social issues, and survival and to clarify the psychologist’s role within the interdisciplinary team.

**Methods:**

Data were retrieved from a prospectively maintained database. From 1st January 2018 to 31st December 2021, 819 consecutive patients were evaluated during EPC consultations, with 753 participants enrolled in the study. The ESAS was administered to each patient before the consultation.

**Results:**

More than half of the patients (385, 57.1%) reported at least one PS, with an ESAS score of ≥4. Specifically, 34.9% reported depression, 28.7% reported anxiety, and 43.2% indicated feeling “not well.” Referring oncologists tended to overestimate the presence of PSs compared to patient self-reports (51.8% versus 41.3%). According to the psychologists’ assessment, 29.2% of participants were found to have depression, and 10.8% of participants had anxiety. Additionally, 31 patients (10.8%) with psychological disorders were diagnosed with an adaptation disorder related to a physical condition. The psychology service engaged 47% of patients, while 18.5% declined psychological support. Patients exhibiting other ESAS symptoms with scores of ≥4 had an increased odds ratio for reporting PSs of ≥4. However, multivariable analysis revealed no significant relationship between PSs and awareness of diagnosis and prognosis.

**Conclusion:**

The systematic use of self-assessment in EPC is essential for understanding patient’s experience, determining whether PSs stem from physical disorders, and prioritizing interventions. Awareness of prognosis does not correlate with increased anxiety and depression in patients. Therefore, EPC is an ideal opportunity to discuss prognosis and facilitate patients’ end-of-life choices early in their care journey.

## 1 Introduction

Over the past 20 years, early palliative care (EPC) has been recommended by leading oncology scientific associations for patients with advanced cancer and has been incorporated into their guidelines, including those from Italy (Cherny et al., 2003; Ferrell et al., 2017; Zagonel et al., 2017). EPC has been found to be strategically helpful in improving overall symptom management and, consequently, the quality of life (QoL) for metastatic cancer patients, with notable benefits for their caregivers’ well-being (Zimmermann et al., 2014; Kaasa et al., 2018; Hui et al., 2022; Borelli et al., 2021). While there is no singular model for EPC consultation, close collaboration between oncologists and interdisciplinary palliative care teams is recommended (Kaasa et al., 2018; Bruera and Hui, 2010; Hui et al., 2015).

The Veneto Institute of Oncology (IOV) is a Comprehensive Cancer Centre where a simultaneous-care outpatient clinic (SCOC) has been established within the Oncology Department since 2014 (Brunello et al., 2022). At this clinic, patients with advanced-stage disease are evaluated by an interdisciplinary team, which includes an oncologist, a palliative care physician, a clinical nutrition specialist, a psychologist, and a nurse navigator, providing EPC alongside anticancer treatment. This approach is implemented regardless of how long patients have been at the center, in accordance with clinical and scientific evidence (Hui et al., 2016).

This fully embedded and value-based model allows us to intercept cancer patients in an advanced stage of disease who need global care. Personalized symptom management, coping and holistic support for patients and caregivers, guidance in decision-making, and shared care planning (SCP) are specific elements of SCOC consultation (Brunello et al., 2022). The SCOC not only identifies patient symptoms but also serves as a critical communication platform, regarded as a ‘time of care’ per Italian law 219/2017 (Law n. 219/2017, 2018). This moment is offered to cancer patients to make them aware of their health status and, through SCP, involve them in end-of-life decisions (Galiano et al., 2022). This embedded model meets internationally agreed criteria for optimizing the early inclusion of palliative care in the patient journey (Hui et al., 2016, 2018) and has been proven to meet the patient’s wishes (Galiano et al., 2024; Bigi et al., 2023a).

In patients with advanced cancer, psychological domains of QoL are frequently considered as outcomes; indeed, patients who report high scores in physical symptoms have an increased risk of developing mood disorders, and they should be screened to provide prompt treatment (Delgado-Guay et al., 2009; Stein et al., 1993). Approximately 50% of patients with advanced cancer meet the criteria for psychopathological disorders, such as adjustment disorders (11–35%) and major depression (5–26%), with serious impact on QoL (Miovic and Block, 2017). Research also shows that a high percentage of advanced cancer patients experience minor or major depression (respectively, 9.6 and 16.5%), adjustment disorder (15.4%), and anxiety disorders (9.8%) (Mitchell et al., 2011). These symptoms can diminish the patient’s ability to cope with both the burden of the illness and its specific symptoms. Additionally, they may negatively impact treatment adherence, increase feelings of isolation, and reduce social interactions. The spectrum of depressive symptoms, although very common, should not be considered a normal response to terminal illness, and timely and appropriate attention to emotional factors is central to end-of-life care (Götze et al., 2014). The impact of psychological problems at this stage can also adversely affect the peace of mind that is needed by the cancer patient, who is aware of his or her prognosis, to participate in the SCP for end-of-life decisions, as also advocated by Italian law (Law n. 219/2017, 2018). Meta-analyses further show that, although effect sizes are small, early palliative care interventions may have more beneficial effects on quality of life and symptom intensity in patients with advanced cancer than in those receiving usual/standard cancer care alone. Effects on mortality and depression are, however, uncertain (Haun et al., 2017). In addition, few studies have currently evaluated patients’ well-being in EPC (Bandieri et al., 2024), and no studies have deepened the role of psychological evaluation during the EPC approach. In the meta-analysis by Fulton et al. (2019), psychological effects were reported in all the studies reviewed, although less than half included a mental health professional in the multidisciplinary team.

Some authors suggest that a different approach is needed: by bringing patients more involved in treatment decisions and SCP, SCOC may have positive effects not only in preventing physical or psychological suffering but also in encouraging SCP for end-of-life choices (Fulton et al., 2019; Hoerger et al., 2018; Bagaragaza et al., 2023; Bigi et al., 2023b). In this perspective, data from 753 patients collected consecutively over 4 years were recently analyzed regarding appropriateness, process, and outcome indicators (Brunello et al., 2022; Galiano et al., 2022).

## 2 Aims

This study aims to do the following:

1. assess the psychological needs identified in a group of patients admitted to the SCOC,
2. assess any correlations between the psychological needs, the awareness of diagnosis and prognosis, other ESAS-detected symptoms, gender, age, social issues, and patient survival, and
3. assess the comparison between self-reported PSs, PS perceived by the oncologist, and PS perceived by the psychologist.

## 3 Materials and methods

### 3.1 Sample

Data were retrieved from the prospectively maintained database of the SCOC. From 1st January 2018 to 31st December 2021, 819 consecutive patients received care at Oncology Unit 1 of the Oncology Department and were evaluated during the SCOC consultation. Of these, 66 patients were no longer undergoing oncological treatment, leaving 753 individuals enrolled in the study. The study was conducted in accordance with the Declaration of Helsinki and was approved by the Veneto Institute of Oncology Ethics Committee (N. 17/2015). The requirement for informed consent was waived due to the retrospective nature of the research and the anonymity of the data.

### 3.2 Setting and measures

Patients with the following characteristics are eligible for SCOC access: (a) advanced disease (locally advanced or metastatic) and (b) under active oncological treatment. Priority access to SCOC is determined by the ‘Request for SCOC’ form completed by the referring oncologist during the patient’s visit (Hui et al., 2015). The SCOC admission criteria are assessed by adding scores that evaluate (a) the Karnofsky Performance Status measure, (b) estimated survival, (c) availability of treatments with an impact on survival, (d) toxicity expected from oncological treatment, (e) presence of socio-family support; (f) the presence of related symptoms (pain, dyspnea, hyporexia, and weight loss) including anxiety and/or depression. No specific criteria have been established to evaluate the presence of anxiety or depression by the referring oncologist.

Prior to the patient-provider consultation, a nurse administers the distress thermometer, and each patient is also asked to complete the Edmonton Symptom Assessment System (ESAS) (Bruera et al., 1991), a validated self-report questionnaire designed to assess the physical and psychological symptoms common in cancer patients. Symptoms are rated on a Likert scale from 1 (no symptoms) to 10 (worst possible outcome). Symptoms assessed are (1) pain, (2) fatigue, (3) nausea, (4) depression, (5) anxiety, (6) drowsiness, (7) well-being, (8) shortness of breath, (9) lack of appetite, and (10) other symptoms. Patients are divided into three groups according to ESAS score: 0–3 (none), 4–6 (moderate), and 7–10 (severe) (Ripamonti et al., 2014; Batra et al., 2022).

When the ESAS is completed, the patient can access SCOC consultation, in which each team member evaluates their area of expertise. In addition to the oncological and palliative care assessment, the psychologist explores the items “anxiety,” “depression,” “not well-being,” and patient awareness of diagnosis and prognosis, and the presence of socio-family support. Moreover, the physician specialized in clinical nutrition assesses the “lack of appetite” and the weight loss and completes the Malnutrition Universal Screening Tool (MUST) to identify patients who are malnourished or are at risk of malnutrition; a score of 0 indicates a low risk of malnutrition, a score of 1 indicates medium risk and a score of ≥2 indicates a high risk (Muscaritoli et al., 2021).

During the SCOC consultation, the interdisciplinary team examines the oncology record and what the referral oncologist reports in the “Request for SCOC” form. At the time of the follow-up visit for anticancer treatment, the referral oncologist assesses the state of the oncological disease, any further treatments available, and the patient’s life expectancy. Each patient is evaluated comprehensively in terms of their overall health, awareness of prognosis, goals of care, and strategies to optimize their decision for the end of life. The psychologist assesses the psychological history and mental state and the presence of anxiety or mood disorders based on the DSM 5 criteria (American Psychiatric Association, 2013).

**Figure 1:**
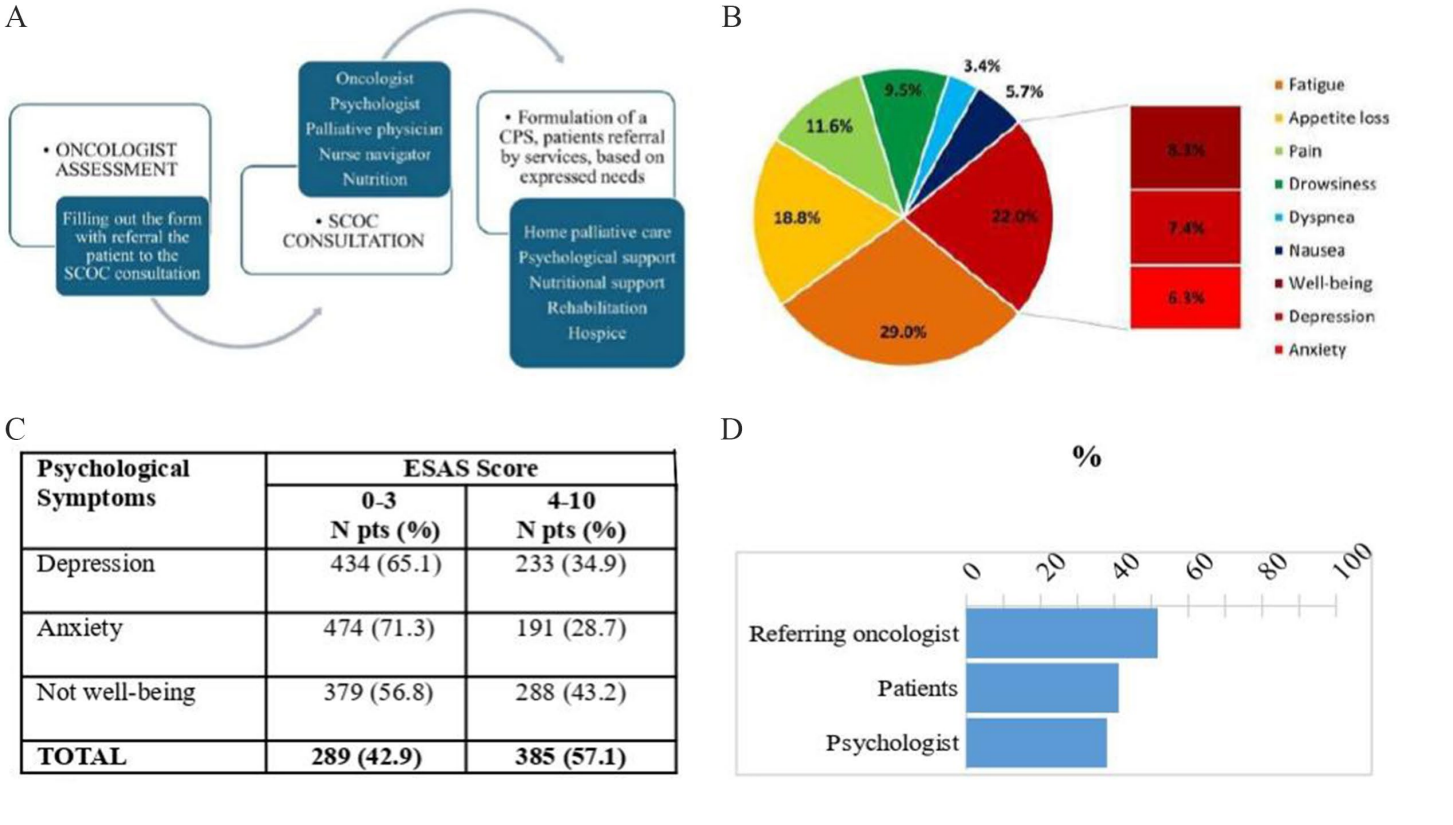
Steps of simultaneous care outpatient clinic (SCOC) for formulation of care plan sharing (CPS) **(A)**; percentage distribution of symptoms with the highest score detected by ESAS **(B)**; psychological symptoms measured by ESAS score **(C)**; comparison of the percentage of anxiety and depression assessed by oncologist (form) by patient (ESAS) and by psychologist **(D)**.

Referrals are then made to other services according to the individual’s physical, mind–body, or social needs, shared with the team (Figure 1A).

### 3.3 Statistical analysis

For analysis, the ESAS symptoms and the MUST variables were dichotomized (ESAS: 0–3, 4–10, and MUST: 0, 1–2).

Descriptive statistics were conducted to report the patient characteristics, ESAS symptoms, and PSs. Cohen’s kappa was used to measure the agreement between the patient’s perception (ESAS score for psychological symptoms), the referring oncologist’s, and the psychologist’s evaluation. For the survival analysis, all patients entered the study on their visit to the SCOC and were followed up until 31 January 2022, or the date of death. Median survival was calculated using the Kaplan–Meier method. Kaplan–Meier survival analysis ensures an accurate survival estimate in the presence of censored data. The date of censure (31st January 2022) is aligned with the articles previously written (Brunello et al., 2022; Galiano et al., 2022). Univariate and multivariable logistic regression models were used to test the association between PSs and explicative variables (gender, age, tumor site, ESAS symptoms, awareness of diagnosis, awareness of prognosis, MUST score, weight loss, family and social issue). The multivariable model was executed using a backward stepwise selection method of sequential variable exclusion. The area under the curve (AUC) was applied to evaluate the goodness of fit of the multivariable model. The level of significance was set at 5%. The analyses were conducted using R software, version 4.3.2, between October and December 2023.

## 4 Results

### 4.1 Symptoms and psychological assessment

Complete descriptives are shown in Table 1. The predominant symptoms in the referral form filled out by the oncologist were appetite loss (74.1%), weight loss (61.9%), pain (56.3%), and psychological disorder (anxiety or depression). These symptoms were recorded by oncologists in 390 patients (51.8%) using the SCOC. All patients filled out the distress thermometer and ESAS before the SCOC visit. Emotional distress has been detected by 69.4% of patients through a distress thermometer. Figure 1B shows the percentage distribution of symptoms with the highest score detected by the ESAS, with the three psychological symptoms (PSs) highlighted. Particularly, after fatigue, which was reported as the symptom with the highest ESAS score in 29% of subjects, PSs represent the second highest area in intensity, reported overall in 22% of patients. As suggested by Battaglia et al. (2020), we use the term “not being well” in place of “well-being.” The feeling of not being well was recorded as the worst ESAS score in 8.3% of subjects, depression in 7.4%, and anxiety in 6.3%. Pain, as the predominant symptom, was reported in only 11.6% of patients.

**Table 1:** Patient characteristics.

Characteristics	*N*	(%)
Total	753	(100)
Gender		
Male	435	(57.8)
Female	318	(42.2)
Age of referral (year)		
Median (IQR)	68	(60–76)
<40	14	(1.9)
41–69	387	(51.4)
>70	352	(46.7)
Tumor site		
Gastrointestinal	566	(75.2)
Genitourinary	113	(15.0)
Other	74	(9.8)
Karnosfsky performance status		
≥70	661	(87.8)
50–60	92	(12.2)
Tumor stage		
Locally advanced	47	(6.2)
Metastatic	684	(90.9)
Missing	22	(2.9)

Figure 1C details the number of subjects with PSs divided by intensity recorded in ESAS. More than half of the patients (385, 57.1%) reported at least one PS with a score of ≥4: depression was reported by 233 (34.9%) of the patients, anxiety by 191 (28.7%) of the patients, and a moderate or severe “not being well” by 288 (43.2%) of the sample. Two hundred and fifteen patients (31.9%) reported through ESAS at least two PSs of intensity greater than or equal to 4. The median overall survival of all patients was 7.3 months (95% CI: 6.5–8.0), while it was 6.5 months (95% CI: 5.7–7.8) in patients with PSs ≥ 4 and 8.3 months (95% CI: 7.2–9.6) in patients with PSs <4 (*p* = 0.2600).

Table 2 shows the assessment of psychological disorders conducted by a psychologist during the SCOC consultation. Out of 753 patients, 286 (38.0%) were found to have psychological issues, with the majority experiencing depression (76.9%) and a smaller percentage experiencing anxiety (28.3%). Some patients exhibited irritability or difficulties with treatment adherence, and a few had a history of psychiatric conditions. Following the SCOC consultation, 99 patients with psychological problems (34.6%) were referred for psychological support by the Institute’s team, while 187 patients (65.4%) did not receive such support. Among those who declined, 37 (19.8%) were already undergoing psychological treatment, 53 (28.3%) refused the intervention, and in 31 patients (16.6%), anxiety and/or depression were attributed to physical symptoms. In 14.4% of cases, the psychological intervention was not feasible due to the patients’ health conditions. Additionally, home palliative care support had already been initiated for 12.3% of patients, while 9.6% of them were referred for psychiatric evaluation for pharmacological treatment, and 16% of the patients were already receiving pharmacological treatments.

**Table 2:** Psychological disorders detected by psychologists during SCOC evaluation.

Psychological problems	*N* (%)	(%)
Total	286	(100)
Depression	220	(76.9)
Anxiety	81	(28.3)
Low compliance	4	(1.4)
Psychiatric history	5	(1.7)
Irritability	3	(1.0)
Other	7	(2.3
Need for psychological intervention		
Yes	99	(34.6)
No	187	(65.4)
Motivation for no psychological intervention^a^		
Already in charge	37	(19.8)
Refusal	53	(28.3)
Psychological adaptation	31	(16.6)
Psyco-pharmacological treatment	30	(16.0)
Health conditions	27	(14.4)
Palliative home care assistance	23	(12.3)
Psychiatric assessment	18	(9.6)
Non-geographic accessibility	1	(0.5)

a Multiple answers.

The agreement among depression and anxiety assessments by referring oncologists, self-reports by patients via ESAS, and evaluations by psychologists during SCOC consultations were analyzed (Figure 1D). The referring oncologists reported 390 times (51.8%) anxiety or depression as symptoms presented by the patients, while the patients reported it 276 times (41.3%) and the psychologist 286 times (38%) (Figure 1D).

### 4.2 Agreement between depression and anxiety symptoms performed by oncologists, patients, and psychologists

The agreement between depression and anxiety symptoms assessed by the referring oncologists, self-reported by the patients by ESAS, and the evaluation performed by the psychologist during SCOC consultation was also analyzed (Table 3). The agreement between the patients and the referring oncologist was equal to 59.5%, with a slight level of agreement (kappa = 0.194). ESAS score and psychologist’s assessment had a moderate level of agreement, equal to 75.3% (kappa = 0.485). With respect to the patient’s age, the correlation between symptoms reported by oncologists and middle-aged adult or elderly patients (more than 70 years old) was slight (agree 59.7%, kappa = 0.191), and by the psychologist was moderate (agree 72% and kappa = 0.457). We found greater concordance between referring oncologists and patients of age < 40 years (agree 69.2% kappa = 0.350, see Table 3).

**Table 3:** Level of agreement between patients with oncologists and psychologists and by patient’s age.

Evaluation	ESAS score for anxiety and depression			Level of agreement		
		0–3	4–10	Agreement (%)	Cohen’s Kappa	Kappa interpretation
All sample						
By psychologist	No	81.7%	33.7%	75.3	0.485	Moderate
	Yes	18.3%	66.3%			
By referring oncologist	No	56.7%	36.6%	59.5	0.194	Slight
	Yes	43.3%	63.4%			
Older adult (≥70 years)						
By psychologist	No	83.4%	38.8%	74.2	0.457	Moderate
	Yes	16.6%	61.2%			
By referring oncologist	No	59.1%	39.5%	59.7	0.191	Slight
	Yes	40.9%	60.5%			
Middle-aged adult (40–69 years)						
By psychologist	No	80.2%	29.5%	76.3	0.507	Moderate
	Yes	19.8%	70.5%			
By referring oncologist	No	54.6%	34.5%	59.0	0.190	Slight
	Yes	45.4%	65.5%			
Young adult (<40 years)						
By psychologist	No	80%	25%	76.9	0.530	Moderate
	Yes	20%	75%			
By referring oncologist	No	60%	25%	69.2	0.350	Fair
	Yes	40%	75%			

### 4.3 Results by univariate and multivariable analysis

Univariate and multivariable analyses were also performed to test relations between PSs and other variables (Table 4). PSs, composed of anxiety, depression, and not-well-being, were statistically associated with the other ESAS symptoms. These variables remained as factors independently associated with PSs in multivariable analysis, while gender lost significantly in the multivariable model. The patients with other ESAS symptoms ≥4 increased the odds ratio (OR) to present PSs ≥ 4. Awareness of diagnosis and prognosis has not been shown to impact PSs, nor have weight loss, MUST value, or family problems.

**Table 4:** Univariate and multivariable models for factors and symptom burden associated with psychological symptoms.

Covariates	Univariate		Multivariable	
	Odds ratio (95% CI)	*p*-value	Adjusted odds ratio (95% CI)	*p*-value
Gender				
Female	References		References	
Male	0.6 (0.4–0.8)	0.0005	0.7 (0.5–1.1)	0.1622
Age				
<70 years	References			
≥ 70 years	1.1 (0.8–1.5)	0.6165	NA	NA
Tumor site				
Others	References			
GI	0.96 (0.7–1.4)	0.8315	NA	NA
Pain				
0–3	References		References	
4–10	1.4 (1.3–1.6)	<0.0001	1.2 (1.1–1.4)	0.0004
Fatigue				
0–3	References		References	
4–10	1.7 (1.5–1.9)	<0.0001	1.4 (1.2–1.6)	<0.0001
Nausea				
0–3	References		References	
4–10	1.4 (1.2–1.5)	<0.0001	1.2 (1.0–1.3)	0.0486
Drowsiness				
0–3	References		References	
4–10	1.6 (1.4–1.7)	<0.0001	1.3 (1.2–1.5)	<0.0001
Appetite loss				
0–3	References		References	
4–10	1.4 (1.3–1.5)	<0.0001	1.1 (1.0–1.3)	0.0220
Dyspnea				
0–3	References		References	
4–10	1.4 (1.3–1.6)	<0.0001	1.3 (1.1–1.5)	0.0027
Awareness of diagnosis				
No	References		References	
Yes	2.7 (0.3–59.0)	0.4131	17.2 (0.6–1011.7)	0.1139
Awareness of prognosis				
No	References		References	
Yes	0.5 (0.1–1.8)	0.3231	0.1 (0.0–1.1)	0.1055
MUST				
0	References			
1–2	1.7 (1.2–2.3)	0.0021	NA	NA
Weight loss				
No	References			
Yes	1.6 (1.1–2.2)	0.0053	NA	NA
Family and social issues				
No				
Yes	1.9 (0.9–4.7)	0.1293	NA	NA

## 5 Discussion

The present study aimed to describe and highlight, in a large group of consecutive patients with metastatic cancer admitted at SCOC consultation, the psychological needs, assess any correlations between the PSs, the symptoms detected by ESAS, the gender and age of the patients, awareness of diagnosis and prognosis, and define the role of psychologist within the interdisciplinary team involved in the SCOC. Shared-approach models for delivering EPC, such as SCOC, provide a unique opportunity to intercept the needs of cancer patients early and share a supportive care program, setting priorities based on patients’ expressed needs (Vanbutsele et al., 2018).

As reported in the literature, our current results show that individuals suffering from advanced cancer experience depression and anxiety (HINZ et al., 2010; Sewtz et al., 2021; Gontijo Garcia et al., 2023). According to a psychologist’s assessment, depression was found in 29.2% (220 of 753 patients) and anxiety in 10.8% (81 of 753) of patients.

A total of 47% of patients were taken in by the psychology service (99 patients at the time of SCOC consultation, and 37 were already in treatment), while some patients met the criteria for a diagnosis of mood or anxiety disorder (n = 18, 9.6%) and were referred for psychiatric evaluation. The proportion of patients who require psychiatric support or are already undergoing psychopharmacological treatment (16.0%) appears to be higher than in previous studies (Fisch et al., 2012). This could be explained by the fact that, in our procedure, the psychologist is included in the SCOC and could be more sensitive in identifying pathologies that require a specialized examination. A total of 31 patients (10.8%) with psychological illnesses have the characteristics of an adaptation disorder linked to a physical condition, which makes the patient refractory to psychological intervention (Teunissen et al., 2007). More than half of the patients (57.1%) presented moderate or severe PSs, as reported by ESAS concomitant fatigue (91.7%), appetite loss (64.9%), drowsiness (56.4%), and pain (55.9%). Interestingly, nausea or other collateral effects of oncologic treatment were found to be less associated with poor mood and “not being well.” These results only partially confirm the literature (Yennurajalingam et al., 2016) because of the varied approaches to these patients in different countries (He et al., 2022; Mejareh et al., 2021; Delgado-Guay et al., 2009).

Collected data allows us to identify a subgroup of patients with high levels of PSs who, however, hardly distinguish between their physical and psychological suffering and, consequently, can help to establish the priority of interventions. Moreover, the cause-and-effect relationship cannot be determined with the current data, i.e., whether PSs aggravate some of the physical symptoms or certain symptoms lead to PSs. It is likely to be a bidirectional relationship. These findings are similar to other studies where ESAS symptoms were intercorrelated, especially anxiety and depression with sleep and other symptoms (He et al., 2022; Delgado-Guay et al., 2009; Narayanan et al., 2022; Hui and Bruera, 2017). Multivariable analysis reports, in fact, a close relation between PSs and other symptoms detected by ESAS. In these situations, it is essential to act as soon as possible to prevent the patient from feeling too compromised and from developing feelings of helplessness and depression. The words of one patient, “*When I have no pain, I forget that I have a tumor*,” turn out to be very significant in this regard.

Another reason supporting the need for early intervention is related to the refusal of psychological support by 18.5% of patients. This suggests that if the patient has not first resolved their physical symptoms, they may not be able to accept psychological help. In these cases, the interdisciplinary approach, such as that offered by SCOC consultation, is a unique opportunity to consider suffering from multiple perspectives and to reach an agreement on the interventions and their priority (Vanbutsele et al., 2018).

We also assessed the correlation in the detection of anxiety and depression between oncologists, patients, and psychologists. Our results suggest that there is a gap between patients’ subjective feelings and what the oncologist recognized during his/her assessment. In particular, the oncologist tends to overestimate the presence of depression or anxiety with respect to what the patient self-reports. Conversely, the correlation between patient (ESAS) and psychologist’s assessment appears to be better (moderate) regardless of the age of the patients. In relation to patients’ age, we found a higher correlation between oncologists’ reporting and younger patients’ self-reporting for depression and anxiety. This is in line with studies suggesting that older people with cancer may be disadvantaged when it comes to oncological treatment (Galvin et al., 2024), also because of implicit ageism biases. The increase in this population in the field of oncology care requires an in-depth understanding of these processes and the possible differences in treatments related to age and other differences among patients.

As expected, the multivariable analysis showed no relationship between PSs and patients’ awareness of diagnosis and prognosis. Many studies demonstrated that awareness of diagnosis and prognosis do not affect PSs (Ryan et al., 2022); this is a significant supporting and confirming aspect that good and honest communication, even if of bad news, is always to be pursued in oncologic settings (Ryan et al., 2022) and strengthens the relationship between team and cancer patient (Sallnow et al., 2022). In our sample, 600 patients (79.6%) were aware of their diagnosis, and 554 (73.6%) were aware of either full or partial prognosis. The psychologist’s role within the SCOC is to detect psychological disorders and help the patient on the path of awareness of prognosis and disease evolution. Awareness about prognosis is a long and personal journey that takes time, and a true caring relationship in which the team, each with their own expertise, helps the patient participate in end-of-life decisions. Recent data from an Italian survey on patients in an advanced stage of cancer reinforces the importance of effective communication strategies between healthcare professionals and patients/caregivers regarding illness, understanding, realistic expectations, and planning for the future (Bigi et al., 2023b). This strategy can disclose patients’ preferences about therapy and outcome, ensuring the possibility of solving doubts or questions regarding end-of-life treatment options. As previously reported, patients given early directives received care at the end of life that was strongly associated with their preferences (Silveira et al., 2010). For people with a life-limiting illness, such as cancer patients, conversations about the fact that they are likely to die from their disease must be sensitively offered throughout their disease course. Italian law 219/2017 declares that “the time of communication between physician and patient represents time of care” and provides the tool of SCP (Law n. 219/2017, 2018).

Moreover, from an organizational perspective in oncology, SCOC consultation is the most appropriate listening space and time to communicate to the patients the evolution of the disease, make them aware of the prognosis, and progressively involve them in end-of-life decisions (Brunello et al., 2022). SCOC appears to be an ideal model for building relationships and implementing effective communication between interdisciplinary teams, patients, and caregivers about illness understanding, realistic expectations, acceptance, goals of care, and planning for the future (Bandieri et al., 2024). This communication venue is highly valued by patients, and the presence of multiple professionals simultaneously is an added value in defining priorities for action and a shared care strategy with the patient, including for future choices (Galiano et al., 2024).

### 5.1 Strengths and limitation

Our study increases information on the psychological status of cancer patients accessing an EPC outpatient clinic. It also draws attention to the role of the psychologist not only for proper assessment of psychological disorders but also for assessing the prognosis awareness necessary to share end-of-life choices. This study certainly has some limitations that should be acknowledged. It is mainly descriptive and largely based on self-reported data. This limitation reflects a context in which psychological assessment is gaining relevance, but outcomes are not always assessed with more precise, sophisticated methods. For example, as for the assessment of depression, a gradient of severity was not registered, only the presence/absence of this disorder. It would be important to understand the complex range of signs of mood deflection better, which is often a gradient and not just a dichotomous variable.

## 6 Conclusion

An interdisciplinary approach such as SCOC consultation is essential for providing comprehensive patient care to metastatic cancer patients. Systematic use of self-assessment tests in clinical practice is crucial for understanding the patient’s experience and prioritizing intervention. The inclusion of a psychologist on the team is essential for appropriately addressing psychological disorders. Awareness of the prognosis does not increase anxiety and depression in the patient and should be the basis for helping the patient in an early sharing of end-of-life choices. The SCOC serves as an ideal forum for facilitating effective communication about prognosis, realistic expectations, acceptance, care goals, and future planning. Finally, although these results may be useful at an organizational level, it is interesting to note that educational and awareness efforts should be addressed to patients who, though suffering, still seem to be reluctant to ask for psychological help.

## 7 Clinical implications

Our data confirm the validity of an integrated model for delivering EPC with psychological evaluation in an interdisciplinary context. This approach ensures a comprehensive assessment of the patient, offering the possibility of early intervention in the patient’s most relevant needs. ESAS remains an essential tool for identifying all patient discomfort and valuing the subjective aspect of the illness and what it entails (Hui and Bruera, 2017). Patient listening time helps to recognize the true causes of psychological distress and contextualize it in the comprehensive support to be offered to the patient, according to the necessary priorities. When the PS is not caused by physical symptoms, it should be treated early to improve the QoL (Smith et al., 2003), and the evaluation of emotional conditions should be carried out by an expert. Healthcare institutions should ensure that a psychologist is always included in the interdisciplinary early palliative care team.

## Data Availability

The raw data supporting the conclusions of this article will be made available by the authors without undue reservation.
